# Ergosta-7,9(11),22-trien-3β-ol Attenuates Inflammatory Responses via Inhibiting MAPK/AP-1 Induced IL-6/JAK/STAT Pathways and Activating Nrf2/HO-1 Signaling in LPS-Stimulated Macrophage-like Cells

**DOI:** 10.3390/antiox10091430

**Published:** 2021-09-08

**Authors:** Yi-Ping Huang, Dar-Ren Chen, Wen-Jen Lin, Yu-Hsien Lin, Jiann-Yeu Chen, Yueh-Hsiung Kuo, Jing-Gung Chung, Te-Chun Hsia, Wen-Tsong Hsieh

**Affiliations:** 1 Department of Physiology, China Medical University, Taichung 404333, Taiwan; yphuang@mail.cmu.edu.tw; 2 Comprehensive Breast Cancer Center, Changhua Christian Hospital, Changhua 50006, Taiwan; darren.chen@cch.org.tw; 3 Graduate Institute of Biomedical Science, China Medical University, Taichung 404333, Taiwan; u104001424@cmu.edu.tw; 4 School of Pharmacy, China Medical University, Taichung 406040, Taiwan; mildds@yahoo.com.tw; 5 Center for Advanced Science and Technology, National Chung Hsing University, Taichung 40227, Taiwan; jiannyeu.chen@nchu.edu.tw; 6 Department of Chinese Pharmaceutical Sciences and Chinese Medicine Resources, China Medical University, Taichung 404333, Taiwan; kuoyh@mail.cmu.edu.tw; 7 Chinese Medicine Research Center, China Medical University, Taichung 404333, Taiwan; 8 Department of Biotechnology, Asia University, Taichung 41354, Taiwan; 9 Department of Biological Science and Technology, China Medical University, Taichung 404333, Taiwan; jgchung@mail.cmu.edu.tw; 10 Department of Respiratory Therapy, China Medical University, Taichung 404333, Taiwan; 11 Department of Internal Medicine, China Medical University Hospital, Taichung 404333, Taiwan; 12 Department of Pharmacology, China Medical University, Taichung 404333, Taiwan

**Keywords:** Ergosta-7,9(11),22-trien-3β-ol (EK100), *Cordyceps militaris*, anti-inflammatory response, MAPK/AP-1 signaling pathways, IL-6/JAK/STAT signaling pathways, Nrf2/ HO-1 signaling pathway, atomic force microscopy (AFM)

## Abstract

Chronic inflammation induces autoimmune disorders and chronic diseases. Several natural products activate nuclear factor erythroid 2-related factor 2 (Nrf2) signaling, attenuating inflammatory responses. Ergosta-7,9(11),22-trien-3β-ol (EK100) isolated from *Cordyceps militaris* showed anti-inflammatory and antioxidative activity, but those mechanisms are still unclear. This study is the first to investigate EK100 on antioxidant Nrf2 relative genes expression in LPS-stimulated macrophage-like cell lines. The results showed that EK100 reduced IL-6 (interleukin-6) and tumor necrosis factor-α production. EK100 also attenuated a mitogen-activated protein kinase/activator protein-1 (MAPK/AP-1) pathway and interleukin-6/Janus kinase/signal transducer and activator of transcription (IL-6/JAK/STAT) pathway in LPS-stimulated cells. Toll-like receptor 4 (TLR4) inhibitor CLI-095 and MAPK inhibitors can synergize the anti-inflammatory response of EK100 in LPS-stimulated cells. Moreover, EK100 activated Nrf2/HO-1 (heme oxygenase-1) signaling in LPS-stimulated murine macrophage-like RAW 264.7 cells, murine microglial BV2 cells, and human monocytic leukemia THP-1 cells. However, Nrf2 small interfering RNA (Nrf2 siRNA) reversed EK100-induced antioxidative proteins expressions. In conclusion, EK100 showed anti-inflammatory responses *via* activating the antioxidative Nrf2/HO-1 signaling and inhibiting TLR4 related MAPK/AP-1 induced IL-6/JAK/STAT pathways in the LPS-stimulated cells *in vitro*. The results suggest EK100 acts as a novel antioxidant with multiple therapeutic targets that can potentially be developed to treat chronic inflammation-related diseases.

## 1. Introduction

Chronic inflammation primes several autoimmune diseases and neurodegenerative disorders, leading to cause disability and mortality worldwide [1]. The other studies described the Kelch-like ECH-associated protein/nuclear factor erythroid 2-related factor 2/antioxidant responsive element (Keap1/Nrf2/ARE) signaling pathways to regulate antioxidant gene expression and inhibit the progression of inflammation [2]. Nrf2 also suppresses pro-inflammatory cytokines interleukin-1β (IL-1β) and interleukin-6 (IL-6) by blocking the transcription factor nuclear factor-kappa B (NF-κB) inflammatory response in macrophage cells [3]. Although Nrf2 elevated ARE expressions such as heme oxygenase-1 (HO-1), catalase (CAT), and superoxide dismutases (SODs), Nrf2 also attenuates the free radicals damage response [4].

MAPK signaling induced the release of tumor necrosis factor-α (TNF-α) and interleukin-6 (IL-6) [5]. Then, IL-6 binding to the transmembrane IL-6 receptor and subsequent activation of Janus kinase (JAK), which is following the phosphorylation of the signal transducer and activator of transcription (STAT) 1/3 and the activated STAT complex will translocate from the cytoplasm to the cellular nucleus initiating transcription of STAT3 target genes [6]. Moreover, JAKs are cytoplasmic tyrosine kinases that could phosphorylate and dimerize the STATs. The phosphorylation and dimerization activate STATs translocated into the cellular nucleus. Furthermore, STAT1/3 binding to specific target DNA triggered inflammatory cytokines of TNF-α, IL-2, IL-6 [7,8]. p38 MAPK regulated transcription factor Nrf2 activation and antioxidative HO-1 expression. However, HO-1 is an ARE and exhibited anti-inflammation responses [9].

*Cordyceps militaris* (CM) was traditionally used in ancient times for anti-inflammatory, antioxidant, and anti-aging responses and revitalizing various body systems [10]. Ergosta-7,9(11),22-trien-3β-ol (EK100) was separated and purified from CM, which interfered with Lipopolysaccharide (LPS) docking to Myeloid differentiation protein-2 (MD-2) in the toll-like receptor 4 (TLR4) attenuate inflammatory effect [11]. Moreover, EK100 inhibited the expression of IL-6, iNOS, and NF-κB [12,13]. EK100 inhibited the release of cytokines such as TNF-α, nitric oxide (NO), IL-1β, and IL-6 expressions [14]. In addition, EK100 increased the endogenous antioxidant expression of CAT and SOD in carrageenan-stimulated mice [15]. However, the relationship between antioxidative and anti-inflammatory effects is still unclear. Thus, this study aims to explore EK100 on antioxidant Nrf2 signaling and inflammatory pathways in LPS-stimulated macrophage-like cell lines.

## 2. Materials and Methods

### 2.1. Materials

CM was obtained from Hsinhai Biotechnology (Taichung, Taiwan). Dimethyl sulfoxide (DMSO), dexamethasone (Dexa), paraformaldehyde, bovine serum albumin (BSA), LPS), sodium dodecyl sulfate-polyacrylamide gel electrophoresis (SDS-PAGE), and 4′,6-diamidino-2-phenylindole (DAPI) were acquired from Sigma-Aldrich (St. Louis, MO, USA). Dulbecco’s Modified Eagle’s Medium (DMEM), DMEM/F12 medium, RPMI1640 medium, penicillin, streptomycin, and fetal bovine serum (FBS), Lipofectamine^TM^ 3000, TAK-242 (CLI-095), TRIzol™ Reagent, SuperScript™ II Reverse Transcriptase, RNaseOUT™ Recombinant RNase Inhibitor, Hoechst 33258, SYBR green, Alexa Fluor 488, and Alexa Fluor 594 were purchased from Invitrogen (Carlsbad, CA, USA). Primary antibodies of PI3K, p-PI3K, Akt, p-Akt, IKK, p-IKK, ERK, p-ERK, JNK, p-JNK, p38, p-p38, c-Jun, c-Fos, Nrf2, p-Nrf2, SOD1, SOD2, CAT, HO-1, STAT1, p-STAT1, STAT3, p-STAT3(727), STAT3, p-STAT3(705), JAK1, p-JAK1, JAK2, p-JAK2, PCNA, and β-actin were obtained from Cell Signaling (Beverly, MA, USA).

### 2.2. Cell Culture

RAW 264.7 murine macrophage-like cells and THP-1 human leukemia monocytic cells were obtained from Food Industry Research and Development Institute (Hsinchu, Taiwan). BV2 murine microglial cells were purchased from American Type Culture Collection (Manassas, VA, USA).

RAW 264.7 cells were incubated in a DMEM medium with 10% FBS and antibiotics (100 units/mL penicillin and 100 µg/mL streptomycin). BV2 cells were cultured in a DMEM/F12 with 10% FBS, 2 mM glutamine, and antibiotics. However, THP-1 cells were cultivated in an RPMI1640 medium with 10% FBS and antibiotics. The cells were treated with phorbol 12-myristate 13-acetate (PMA) for 24–48 h to stimulate macrophage differentiation before experiments. All cells were hatched in the incubator at 37 °C containing 5% CO_2_.

### 2.3. Atomic Force Microscopy (AFM) Assay

AFM assay was used to explore three-dimensional morphological information of anti-inflammatory effects as described previously [16]. In brief, RAW 264.7 cells (5 × 10^4^ cells/mL) were cultured on the glass coverslips in a 6-well plate. Cells were treated with 80 μM EK100 or 2 μM Dexa 1 h before 100 ng/mL LPS was stimulated for 24 h. Then, 4% paraformaldehyde was added to fix the cells. The AFM probe was APP-Nano ACTA series whose tip and cantilever spring constant radius. Furthermore, the cell-binding force of AFM was analyzed with NanoScope (Bruker Co., Santa Barbara, CA, USA).

### 2.4. Enzyme-Linked Immunosorbent Assay (ELISA)

Measured the creation of cytokines using ELISA assay as described previously [17]. In brief, cells were treated with 0, 10, 20, 40, and 80 μM EK100 for 1 h before being incubated with LPS in various periods. Then, the cell culture supernatant was collected and determined for cytokines levels using ELISA kits (IL-6 and TNF-α) by Micro-Reader EPOCH2 plate reader (BioTek, Winooski, VT, USA).

### 2.5. Quantitative Polymerase Chain Reaction Assay (qPCR)

Analyzed RNA expression was using qPCR assay as described previously [18]. Briefly, RAW 264.7 cells (1.5 × 10^5^ cells/well) were cultured in 6-well plates. 0, 10, 20, 40, and 80 μM EK100 were treated before LPS treatment. TRIzol reagent with SuperScript™ II Reverse Transcriptase and RNaseOUT™ Recombinant RNase Inhibitor extracted RNA. The RNA which was being collected was transformed into cDNA by using cDNA kits (Roche, Mannheim, Germany). The following PCR primer sequences were used: IL-6_ forward (F): 5′-CCGGAGAGGAGACTTCACAG-3′, and IL-6_reverse (R): 5′-TCCACGATTTCCCAGAGAAC-3′ (Sequence ID: NM_013693.3); TNF-α_F: 5′-TCAGCCTCTTCTCATTCCTG-3′, and TNF-α_R: 5′-TGAAGAGAACCTGGGAGTAG-3′ (Sequence ID: NM_013693.3); GAPDH_F: 5′-GGCCTTCCGTGTTCCTACC-3′, GAPDH_R: 5′-TGCCTGCTTCACCACCTTC-3′ (Sequence ID: BC023196.2). The thermal cycler parameters were followed by 40 cycles of 95 °C for 10 s and 60 °C for 30 s. StepOne Plus Real-Time PCR Systems (Applied Biosystems, Carlsbad, CA, USA) were applied to PCR reactions, which also operated using SYBR green working solution. The following steps about thermal cycler parameters were used 95 °C for 10 min, followed by 40 cycles of 95 °C for 10 s, and 60 °C for 30 s.

### 2.6. Western Blotting Analysis (WB)

WB was used for analytical performance in immunogenetics to detect specific proteins described previously [19]. In brief, cells were treated with 0, 10, 20, 40, and 80 μM EK100 1 h before LPS. Proteins were extracted by PRO-PREP™ and then separated by 8-12% SDS-PAGE. Proteins were transferred from gel to polyvinylidene fluoride (PVDF) membranes (Millipore Co. Billerica, MA, USA) and blocked with 5% BSA. Then probed with the primary antibodies overnight at 4 °C before incubated with horseradish peroxidase (HRP) conjugated secondary antibody. The antibody detection reaction was performed with enhanced chemiluminescence (ECL) (Amersham, Piscataway, NJ, USA). The antibodies were captured using a biomolecular imager (Las 4000 mini, GE, Pittsburgh, PA, USA).

### 2.7. Immunofluorescence Assay (IF)

IF assay was used for visualized the specificity of antibodies with fluorescent dyes in the cells. Therefore, it allows visualization of the target proteins distribution through the sample under a fluorescence microscope, as described previously [20]. In brief, cells were incubated in a confocal laser dish (500 cells/dish) for 16 h and treated with 80 μM EK100 or 2 μM Dexa before being incubated with LPS. Cells were fixed with 4% paraformaldehyde, then permeabilized with 0.25% Triton X-100. It blocks nonspecific binding by 5% PBS-BSA, probes with the primary antibodies, and labels a secondary antibody with IgG Alexa Fluor 488 or Alexa Fluor 594. After that, the nuclei were stained with DAPI gel (1 μg/mL) in 1% BSA for 20 min at 37 °C in the darkness. IF staining images were visualized with an SP2/SP8X Confocal Spectral Microscope (Leica Microsystems, Wetzlar, Germany).

### 2.8. Nrf2 siRNA Transfection Assay

Nrf2 siRNA transfection assay was used to analyze the Nrf2 antioxidation activity, as described previously [18]. In brief, RAW 264.7 cells were cultured in 6-well plates (2 × 10^5^ cells/well). Transfection of DNA fragment encoding Nrf2 siRNA or Nrf2-negative control siRNA was performed using Lipofectamine^TM^ 3000 (Invitrogen). Nfe2l2 Mouse siRNA Oligo Duplex was used for transfection of small interfering RNA (siRNA). Nrf2 siRNA to knockdown endogenous Nrf2, confirming the protocol formulated by the manufacturer (Invitrogen). After 24–48 h, EK100 and LPS mixture was added to the transfected cells for 18 h, followed by WB and the other analyses.

### 2.9. Statistical Analysis

All experimental data were demonstrated as the mean ± SEM obtained from 3 individual experiments, and experiments were conducted in triplicates (*n* = 3). Statistical significance was performed using one-way analysis of variance (ANOVA) followed by Tukey’s honest significant difference (HSD) test or the Student’s two-tailed *t*-test to determine the statistical significance via SPSS17.0 software system (IBM, Chicago, IL). Differences were measured statistically significant at the level when *p*-value < 0.05.

## 3. Results

### 3.1. EK100 Inhibited IL-6 and TNF-α Released in LPS-Stimulated RAW 264.7 Cells

**Figure 1:**
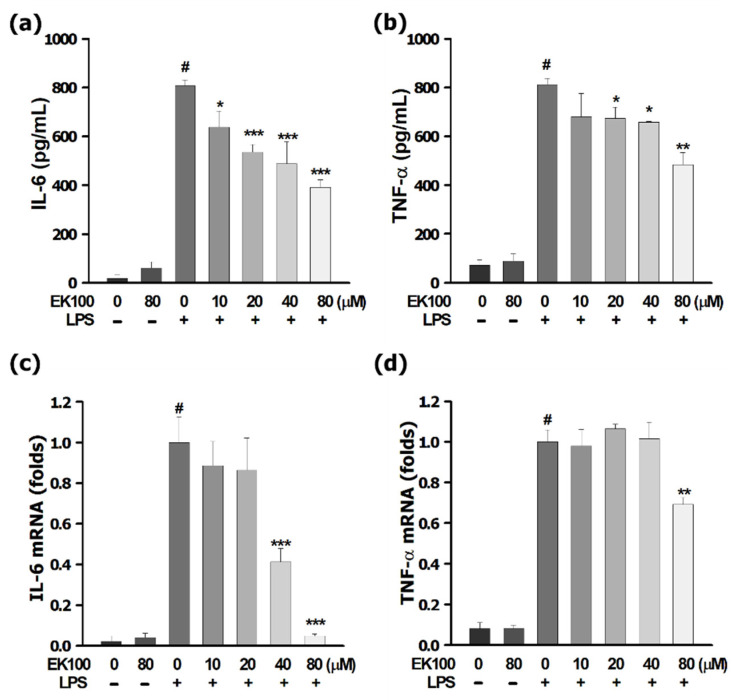
EK100 inhibited IL-6 and TNF-α released in LPS-stimulated RAW 264.7 cells. RAW 264.7 cells were treated with 0, 10, 20, 40, and 80 µM EK100 for 1 h before being stimulated with 100 ng/mL LPS for 24 h. Then the suspension media was separated from the remaining cells. In the suspension media, we detected the cytokines productions of IL-6 (**a**) and TNF-α (**b**) by using the specific ELISA kit, respectively. It extracted and analyzed IL-6 (**c**) and TNF-α (**d**) mRNA in the remaining cells by using *q*PCR assay as described in Materials and Methods. Data are presented as the means ± SEM of three independent experiments (*n* = 3). # *p* < 0.05 compared to the control group, * *p* < 0.05, ** *p* < 0.01, and *** *p* < 0.001 compared to LPS alone group.

The results indicated that LPS induced the production of IL-6 and TNF-α in RAW 264.7 cells. Compared to the LPS group, EK100 at 80 μM decreased inflammatory cytokines of IL-6 and TNF-α, respectively. EK100 reduced the ratio of inflammatory cytokines of IL-6 (Figure 1a) and TNF-α (Figure 1b), respectively. Moreover, LPS increased the cytokine production of IL-6 and TNF-α, and EK100 also reduced the mRNA expression of TNF-α and IL-6. In the production of pro-inflammatory cytokines expression of mRNA, EK100 significantly inhibited mRNA of IL-6 (Figure 1c) and TNF-α (Figure 1d), respectively. Those results showed EK100 suppressed LPS-stimulated cytokines released from IL-6 and TNF-α in RAW 264.7 cells.

### 3.2. EK100 Prevented the Morphological Change in LPS-Stimulated RAW 264.7 Cells

**Figure 2:**
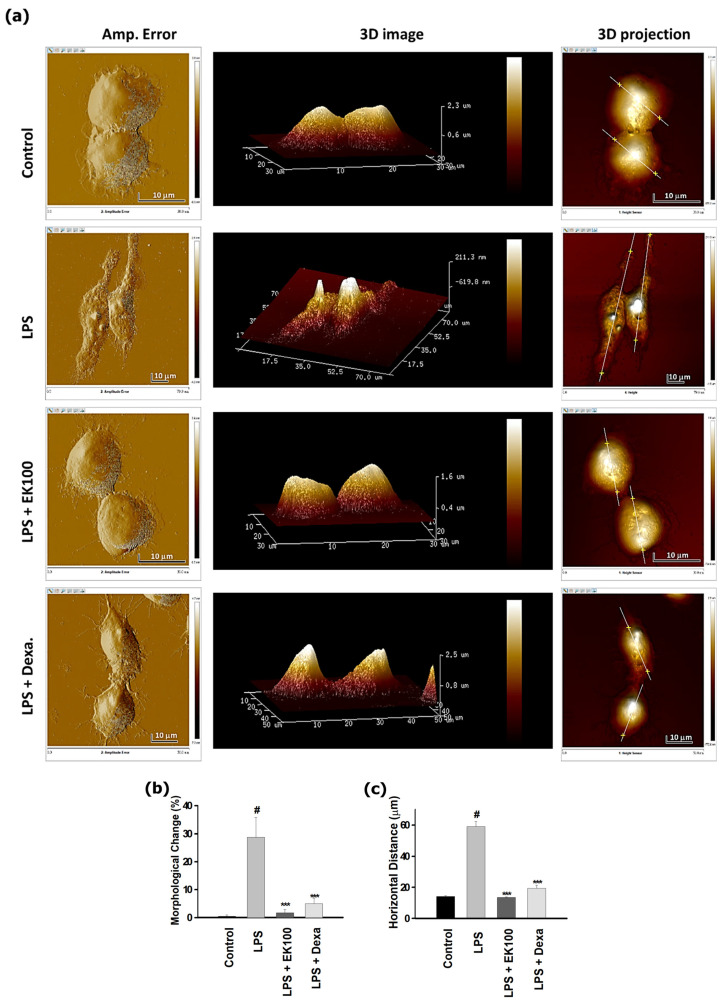
EK100 prevented the morphological change in LPS-stimulated RAW 264.7 cells. (**a**) Cells were treated with 80 μM EK100 or Dexa (2 μM) for 1 h and stimulated with 100 ng/mL LPS for 24 h. The dendritic transformation assay and surface ultrastructural morphological change by AFM assay, including amplitude error, three-dimensional (3D) images, 3D projection, and horizontal distances as designated and described in Materials and Methods. Those analysis data from AFM assay of the ratio of morphological change (**b**) and the horizontal distance (**c**) were analyzed using NanoScope analysis software. All data calculated in the cells were presented as the mean ± SEM of three independent experiments (*n* = 3). # *p* < 0.05 compared with the control group and *** *p* < 0.001 as compared with LPS alone group.

Almost 23.65% of the LPS-stimulated only group showed dendritic morphological transition change and lamellipodia in the AFM assay. Conversely, EK100 and Dexa presented significantly reverse LPS-stimulated morphological adaptation to the distinctive oval shape, with the smooth cell surface and converse (Figure 2a). The morphological change (length/width > 1.5) of AFM assay in the control group was below 0.5% in RAW 264.7 cells. However, after LPS-stimulated, the morphological change in the only LPS-induced group increased to 28.7 ± 7.1%, but the EK100 group significantly reduced to 1.7 ± 1.2%, and the Dexa group reduced to 5.0 ± 2.0% (Figure 2b). Moreover, the horizontal distance in the control group was 13.6 ± 0.5 μm. However, the LPS-induced alone group increased to 59.0 ± 3.3 μm, the EK100 group significantly reduced to 13.6 ± 0.4 μm, and the Dexa group reduced to 19.4 ± 1.97 μm (Figure 2c). Analysis of variance with AFM assay showed that EK100 and Dexa could reverse LPS-stimulated morphological changes in RAW 264.7 cells.

### 3.3. EK100 Suppressed the MAPK/AP-1 Pathways in LPS-Stimulated RAW 264.7 Cells

**Figure 3:**
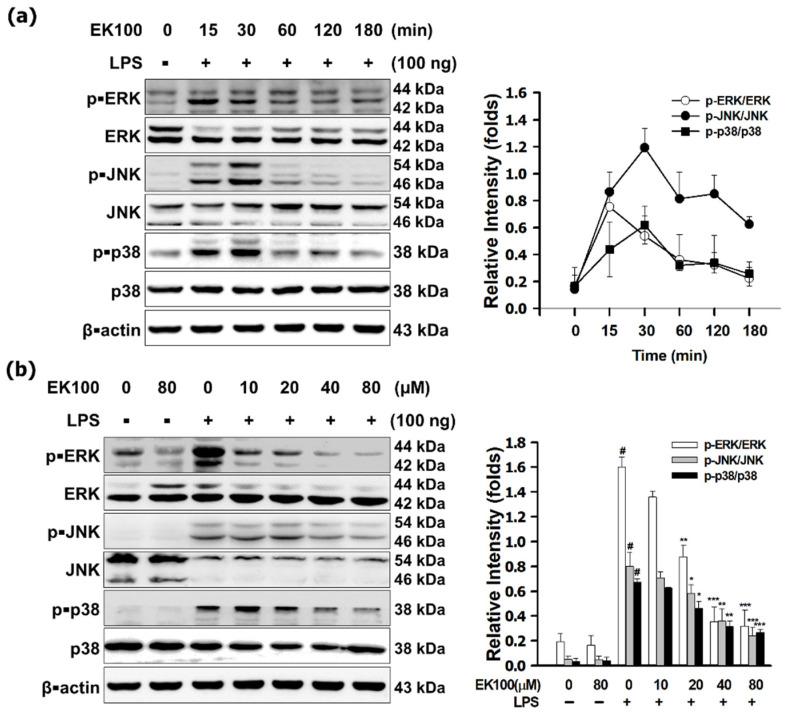

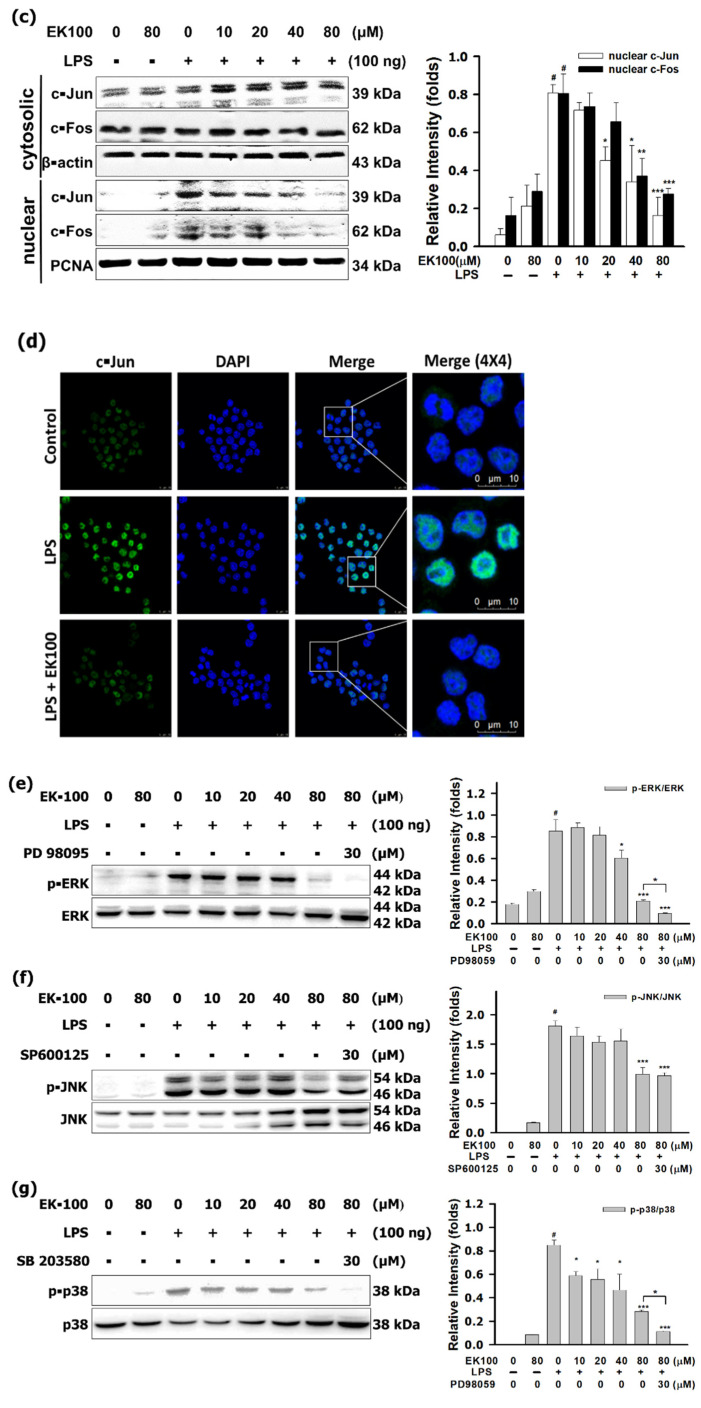

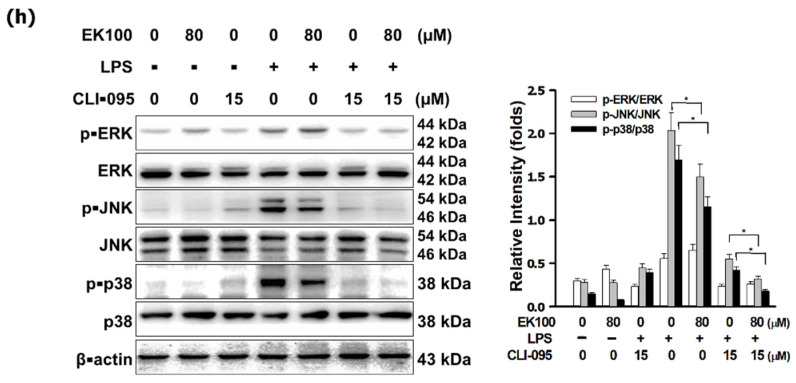
EK100 suppressed the MAPK/AP-1 pathways in LPS-stimulated RAW 264.7 cells. (**a**) Cells were treated with 0, 10, 20, 40, and 80 µM EK100 and then treated with 100 ng/mL LPS to evaluate the protein expression maximum peaks of p-ERK, p-JNK, and p-p38 in LPS-stimulated RAW 264.7 cells. (**b**) Cells were treated with 0, 10, 20, 40, and 80 µM EK100 for 1 h before being stimulated with LPS for 30 min. Then used WB analyzed MAPK protein levels (**c**). The protein expressions of transcription factor c-Jun and c-Fos in cytoplasm and nucleus were measured by WB. (**d**) Cells were treated with 80 μM EK100 for 1 h before being stimulated with LPS for 2 h. The localization and expression of c-Jun were measured by IF staining as designated in Materials and Methods. Cells were treated with EK100 and MAPK inhibitor (PD98095, SP600125, and SB203580) and TLR4 (CLI-095) for 1 h before being stimulated with LPS for 30 min. The protein expressions of p-ERK (**e**), p-JNK (**f**), p-p38 (**g**), and p-MAPKs (**h**) were detected by WB. Data presented as folds means ± SEM compared with β-actin in the cellular cytoplasm or PCNA in the cellular nucleus of three independent experiments (*n* = 3). # *p* < 0.05 compared with the control group, * *p* < 0.05, ** *p* < 0.01, and *** *p* < 0.001 compared with LPS alone or group no EK100 treated group.

As shown in Figure 3a, LPS increased the maximum pro-inflammatory mediator expression of phosphorylation of MAPKs; within 15–30 min LPS-induced phosphorylated MAPKs. This study indicates that EK100 attenuated phosphorylated MAPKs significantly. Compared with the LPS-induced only group, 80 μM EK100 suppressed the phosphorylated extracellular signal-regulated kinase (ERK) to 0.19 ± 0.03 folds, c-Jun N-terminal kinase (JNK) to 0.29 ± 0.03 folds, and p38 MAPK (P38) to 0.39 ± 0.02 folds (Figure 3b). Consequently, LPS-induced transcription factor AP-1 translocated into the cellular nucleus from the cytoplasm in the cells, and EK100 attenuated c-Jun and c-Fos significantly. Compared with the LPS-induced only group, 80 μM EK100 inhibited c-Jun level to 0.2 ± 0.05 folds and c-Fos level to 0.34 ± 0.02 folds, respectively (Figure 3c). IF staining also showed EK100 inhibited the expression of c-Jun in the cellular nucleus (Figure 3d). However, ERK inhibitor PD98095-synergized EK100 attenuated the phosphorylated ERK1/2 (Figure 3e). JNK inhibitor SP600125 did not influence that EK100 attenuated the phosphorylation of JNK in LPS stimulated cells (Figure 3f). p38 inhibitor SB203580-synergized EK100 attenuated the phosphorylated p38 (Figure 3g). In RAW 264.7 cells, LPS also induced the phosphorylated ERK1/2, JNK, and p38. EK100 reduced phosphorylated ERK1/2, JNK, and p38. Moreover, TLR4 inhibitor CLI-095-synergized EK100 reduced the phosphorylated ERK1/2, JNK, and p38 (Figure 3h). The results indicated that EK100 reduced LPS-stimulated phosphorylated ERK1/2, JNK, and p38 and inhibited the translocation and activation of c-Jun and c-Fos. These results further supported that EK100 attenuated the TLR4 signaling induced MAPK/AP-1 signaling pathways in RAW 264.7 cells.

### 3.4. EK100 Inhibited the JAKs/STATs Pathways in LPS-Stimulated RAW 264.7 Cells

**Figure 4:**
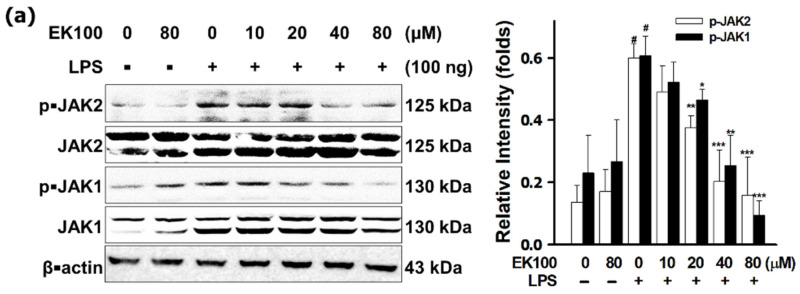

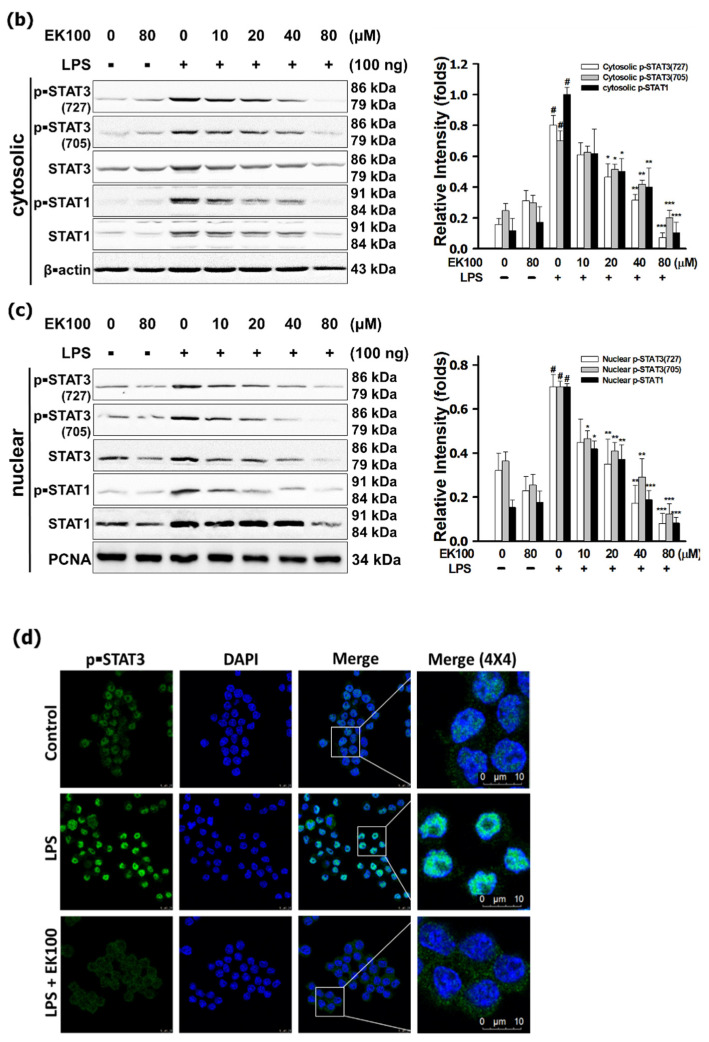
EK100 suppressed the MAPK/AP-1 pathways in LPS-stimulated RAW 264.7 cells. Cells were treated with 0, 10, 20, 40, and 80 µM EK100 for 1 h before being stimulated with 100 ng/mL LPS for 6 h. (**a**) The phosphorylation protein expressions JAK1/2 were analyzed. (**b**) The phosphorylation protein expressions p-STAT1 (705), p-STAT3 (727), and p-STAT3 (705) in the cellular cytoplasm were analyzed. (**c**) The phosphorylation and protein expression in the cellular nucleus of p-STAT1 (705), p-STAT3 (727), and p-STAT3 (705) were analyzed. (**d**) Cells were treated with 80 μM EK100 for 1 h before being stimulated with LPS for 6 h. p-STAT (Tyr705) was measured with IF staining. All experiments were designated and described in Materials and Methods. Data presented as folds means ± SEM compared with β-actin in the cellular cytoplasm or PCNA in the cellular nucleus of three independent experiments (*n* = 3). # *p* < 0.05 compared with the control group, * *p* < 0.05, ** *p* < 0.01, and *** *p* < 0.001 compared with LPS alone group.

The results displayed that LPS stimulated phosphorylated JAK1/2 and EK100 attenuated p-JAK1/2 significantly (Figure 4a). LPS also stimulated phosphorylated STAT1/3 and EK100 attenuated p-STAT1, p-STAT3 (727), and p-STAT3 (705) significantly in the cytoplasm (Figure 4b). Consequently, EK100 also reduced LPS-stimulated protein expression and translocation of p-STAT1 and p-STAT3 in the cellular nucleus (Figure 4c). Furthermore, 80 μM EK100 inhibited the ratio of protein expression of nuclear transcription factor p-STAT1 level to 0.11 ± 0.03 folds, p-STAT3 (727) level to 0.17 ± 0.03 folds, and p-STAT3 (705) level to 0.11 ± 0.02 folds, respectively (Figure 4c). As shown in Figure 4d, IF staining assay also showed that LPS-induced p-STAT3 (705) translocated into the cellular nucleus, and EK100 prevented the nuclear translocation of p-STAT3 (705) into the cellular nucleus significantly. These findings indicate that EK100 attenuated the transcription factor proteins translocation and activation of p-STAT1 and p-STAT3 in RAW 264.7 cells.

### 3.5. EK100 Activated the Nrf2/HO-1 Signaling Pathway in LPS-Stimulated Macrophage-like Cells

**Figure 5:**
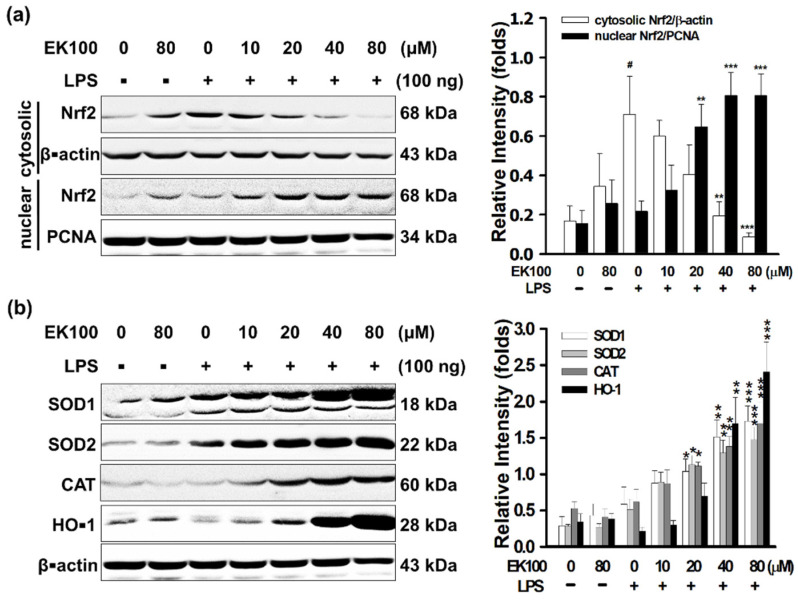

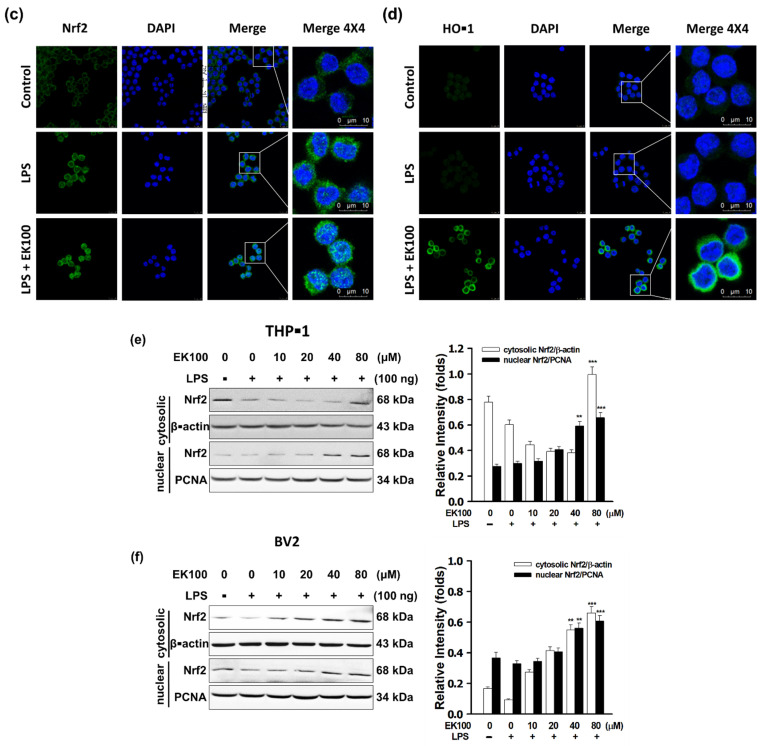

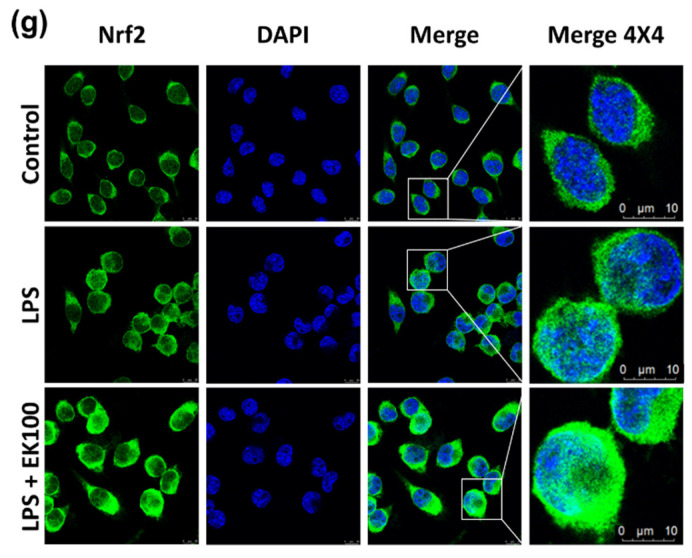
EK100 activated the Nrf2/HO-1 signaling pathway in LPS-stimulated macrophage-like cells. Cells were treated with 0, 10, 20, 40, and 80 µM EK100 before being stimulated with 100 ng/mL LPS. In RAW 264.7 cells: (**a**) The protein levels of the antioxidative transcription factor Nrf2 in the cytoplasm and cellular nucleus were measured by WB. (**b**) The protein expressions of HO-1, SOD1, SOD2, and CAT were measured. (**c**) Representative images showing the effect of 80 μM EK100 on Nrf2 protein expression were measured by IF staining. (**d**) Illustrative images of IF staining were showing the effect of 80 μM EK100 on HO-1 protein expression. In THP-1 cells: (**e**) Cells were treated with 0, 10, 20, 40, and 80 µM EK100 before being stimulated with LPS for 24 h. WB analyzed the protein levels of Nrf2 in the cellular cytoplasm and cellular nucleus. In BV2 cells: (**f**) Cells were treated with 0, 10, 20, 40, and 80 µM EK100 before being stimulated with LPS for 24 h. The protein levels of Nrf2 in the cellular cytoplasm and cellular nucleus were measured by WB. (**g**) Illustrative images of IF staining showed the effect of 80 μM EK100 on Nrf2 protein expression in BV2 cells. In RAW 264.7 cells: All the experiments were designated in Materials and Methods. All results were expressed as folds mean ± SEM compared with β-actin in the cytoplasm or PCNA in the cellular nucleus of three independent experiments (*n* = 3). # *p* < 0.05 compared with the control group, * *p* < 0.05, **p < 0.01, and *** *p* < 0.001 compared with the LPS alone group.

In RAW 264.7 cells, EK100 stimulated antioxidative Nrf2 protein expression significantly in the cellular nucleus of RAW 264.7 cells. At 80 μM EK100, and compared with the LPS group, Nrf2 protein expression increased from 0.22 ± 0.02 to 0.81 ± 0.06 folds in the cellular nucleus (Figure 5a). Simulation results indicated that EK100 stimulated Nrf2-relative antioxidative protein expression, including HO-1, SOD1, SOD2, and CAT, was significant. At 80 μM EK100, and compared with the LPS group, EK100 increased the antioxidation protein level of HO-1 protein from 0.22 ± 0.08 to 4.20 ± 0.80 folds, the SOD1 protein level from 0.58 ± 0.23 to 1.72 ± 0.21 folds, the SOD2 protein level from 0.47 ± 0.17 to 1.47 ± 0.17 folds, and the CAT protein level from 0.61 ± 0.17 to 1.70 ± 0.29 folds, respectively (Figure 5b). EK100 stimulated the antioxidative transcript factor Nrf2 translocated into the cellular nucleus (Figure 5c). EK100 also promoted the antioxidative proteins of HO-1 expression but only in the cellular cytoplasm (Figure 5d). These results indicated that EK100 induced the transcription factor Nrf2 translocated into the cellular nucleus. Nrf2 activated the antioxidative relative gene expressions of HO-1, SOD1, SOD2, and CAT in RAW 264.7 cells.

In THP-1 cells, EK100 stimulated antioxidative Nrf2 in the cellular nucleus significantly. At 80 μM EK100 treatment, Nrf2 protein level increased from 0.31 ± 0.02 to 0.65 ± 0.02 folds in the cellular nucleus (Figure 5e).

In BV2 cells, EK100 also stimulated antioxidative Nrf2 in the cellular nucleus significantly. At 80 μM EK100 treatment, Nrf2 protein level increased from 0.34 ± 0.02 to 0.60 ± 0.02 folds in the cellular nucleus (Figure 5f). Moreover, in BV2 cells with IF staining assay, we found that EK100 also stimulated the antioxidative transcript factor Nrf2 in the cellular nucleus and cytoplasm in LPS-stimulated BV2 cells (Figure 5g).

The results indicated that EK100 elevated the antioxidative transcription factor Nrf2 translocated into the cellular nucleus. EK100 also activated Nrf2/HO-1 signaling pathway in murine macrophage-like RAW 264.7 cells, human leukemia monocytic THP-1 cells, and murine microglial BV2 cells.

### 3.6. Nrf2 siRNA Reversed EK100 Activated the Nrf2/HO-1 Pathway in LPS-Stimulated Cells

**Figure 6:**
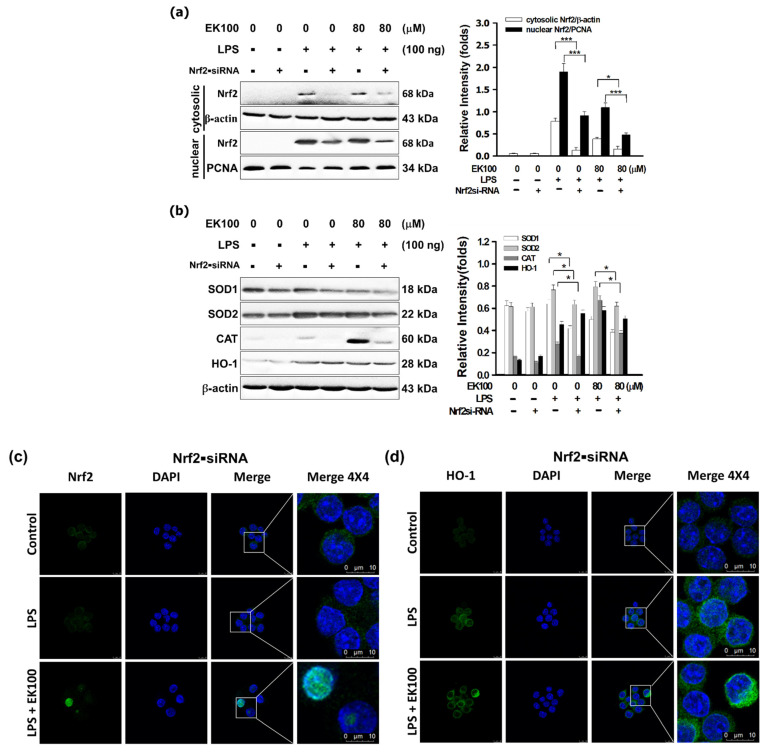
Nrf2 siRNA reversed EK100 activated the Nrf2/HO-1 pathway in LPS-stimulated cells. (**a**) Nrf2 siRNA or Nrf2-negative control siRNA was transfected into cells for 24 h and was collected. Cells were treated with 80 μM EK100 and 15 μM CLI-095 for 1 h before being stimulated with LPS for 6 h. The protein levels of Nrf2 in the cellular cytoplasm and cellular nucleus were analyzed by WB assay. (**b**) Nrf2 siRNA or Nrf2-negative control siRNA was transfected into cells for 24 h and was collected. Cells were treated with 80 μM EK100 and 15 μM CLI-095 for 1 h before being stimulated with LPS for 24 h. WB analyzed the protein expressions of HO-1, SOD1, SOD2, and CAT. (**c**) Nrf2 siRNA or Nrf2-negative control siRNA was transfected into cells for 24 h and was collected. Representative images showing the effect of 80 μM EK100 on Nrf2 protein expression were measured by IF staining in LPS-stimulated RAW 264.7 cells. (**d**) Nrf2 siRNA or Nrf2-negative control siRNA was transfected into cells for 24 h and was collected. Illustrative images of IF staining showed the effect of 80 μM EK100 on HO-1 protein expression in LPS-stimulated RAW 264.7 cells. All the experiments were designated in Materials and Methods. All results were expressed as folds mean ± SEM compared with β-actin in the cytoplasm or PCNA in the cellular nucleus of three independent experiments (*n* = 3). # *p* < 0.05 compared with the control group, * *p* < 0.05 and *** *p* < 0.001 compared with the LPS alone group or no Nrf2 siRNA treated group.

Although EK100 inhibited the Nrf2 protein expression in the cytoplasm, EK100 stimulated antioxidative Nrf2 protein expression significantly in the cellular nucleus of RAW 264.7 cells. Moreover, Nrf2 siRNA attenuated with or without EK100 stimulated antioxidative Nrf2 protein expression in the cellular nucleus and cytoplasm. At 80 μM EK100 with LPS promoted and compared with or without pretreated Nrf2 siRNA, the antioxidative protein level of Nrf2 protein level decreased from 1.09 ± 0.06 to 0.47 ± 0.02 folds in the cellular nucleus of RAW 264.7 cells (Figure 6a). Simulation results indicated that Nrf2 siRNA attenuated with EK100 stimulated the protein expressions of HO-1, SOD1, SOD2, and CAT, respectively. At 80 μM EK100 with LPS stimulated, and compared with pretreated Nrf2 siRNA, the antioxidative protein levels of HO-1 protein decreased from 0.58 ± 0.03 to 0.50 ± 0.03 folds, SOD1 protein level reduced from 0.50 ± 0.02 to 0.38 ± 0.02 folds, and SOD2 protein level decreased from 0.79 ± 0.04 to 0.62 ± 0.03 folds. CAT protein levels decreased from 0.67 ± 0.03 to 0.37 ± 0.02 folds in RAW 264.7 cells, respectively (Figure 6b). Moreover, in the IF staining assay, we found that Nrf2 siRNA reversed EK100 induced the antioxidative transcript factor Nrf2 protein expression in the cellular nucleus (Figure 6c) and HO-1 protein expression cellular cytoplasm (Figure 6d) in LPS-stimulated RAW 264.7 cells. The results displayed that Nrf2 siRNA reversed EK100 induced the overexpression of the Nrf2/HO-1 antioxidative signaling pathway.

## 4. Discussion

Chronic inflammation is critical for survival during bodily injury and infection that causes disability and mortality for patients. Accordingly, the MAPK/AP-1 pathway is prominent in releasing cytokines of IL-1, IL-6, IL-8, and TNF-α and activated the phosphorylated STATs [21,22]. Moreover, LPS-induced TLR4, NF-κB, and MAPK signaling pathways are required to control the regulation of IL-6 expression [23]. The present studies indicated that LPS-stimulated inflammatory cytokines release IL-6 and TNF-α in RAW 264.7 cells. However, EK100 inhibited the release and the mRNA expression of the cytokines of IL-6 and TNF-α in LPS-stimulated RAW 264.7 cells (Figure 1a–d). The AFM is a novel nanotool that shows the height distribution of the cell membrane topography and reflects the complexity of cell membrane ultrastructure images that are beneficial for investigating potential targets for anti-inflammatory drugs on native macrophages [16,24]. The LPS-stimulated only group showed dendritic morphological transition change and lamellipodia; EK100 presented significantly reverse LPS-stimulated morphological change to the distinctive oval shape with the smooth cell surface and converse. Herein, EK100 and Dexa reduced the morphological change and cellular size in LPS-stimulated RAW 264.7 cells. Thus, the AFM assay in Figure 2a–c provided evidence and confirmed that EK100 prevented inflammatory response and the dendritic transformation in LPS-stimulated RAW 264.7 cells *in vitro*.

TLR4 activates the MAPK/IKK pathways to induce inflammatory transcription factor NF-κB, and AP-1 translocates into the nucleus and increases the release of TNF-α and IL-6 [25,26]. Phosphatidylinositol 3-kinase B (PI3K/Akt) activated the MAPK/AP-1 signaling [27]. AP-1 includes c-Fos and c-Jun heterodimers, the transcription factors that mediate many biological processes [28]. The activation of transcription factors c-Fos and c-Jun has been shown to stimulate iNOS and COX-2 expression; however, c-Jun can activate Nrf2-induced transcription, and c-Fos can suppress Nrf2-induced transcription [29,30,31]. EK100 interfered with LPS docking to TLR4/MD-2 co-Receptors to attenuate the inflammatory cytokines NO and PGE_2_ releases [11]. As shown in Figure 3a, LPS increased the pro-inflammatory mediator expression of MAPKs. However, EK100 attenuated the phosphorylated ERK1/2, JNK, and p38 (Figure 3b). EK100 inhibited c-Jun and c-Fos translocated into the cellular nucleus from the cytoplasm in the cells (Figure 3c). IF staining also showed EK100 inhibited the expression of c-Jun in the cellular nucleus (Figure 3d). These results noted that EK100 prevented LPS-stimulated phosphorylated ERK1/2, JNK, and p38 and inhibited the translocation of c-Jun and c-Fos in RAW 264.7 cells (Figure 3e–g). TLR4 inhibitor CLI-095 and MAPK inhibitors synergized EK100 attenuated MAPK/AP-1 signaling pathways (Figure 3h). The results supported and confirmed that EK100 attenuated the TLR4 signaling-related MAPK/AP-1 inflammatory signaling pathways in LPS-stimulated RAW 264.7 cells.

Elevated phosphorylated STAT (p-STAT) raised chronic inflammatory activity [1]. Activation of STAT3 and NF-κB elevated IL-6-mediated overexpression of COX-2 in chronic inflammatory diseases [32]. p38 regulates the IL-6-induced transcriptional activation of STAT3 during the inflammatory process and keeps cells alive [33,34]. Herein, it was observed that EK100 attenuated LPS-stimulated protein expression of p-JAKs (Figure 4a) and translocation of p-STAT1, p-STAT3 (727), and p-STAT3 (705) in the cellular nucleus (Figure 4b,c). Furthermore, EK100 inhibited LPS-stimulated nuclear transcription factors p-STAT3 (727) expression were examined with IF staining (Figure 4d). Based on these findings, it is indicated that EK100 attenuated IL-6 activated a JAK/STAT inflammatory and stress signaling pathway in LPS-stimulated RAW 264.7 cells.

Nrf2 was reflected as the cytoprotective factor regulating the antioxidative, anti-inflammatory, and detoxifying activities [35]. The antioxidant mechanisms of the Keap1/Nrf2 antioxidant response element (ARE) were to eliminate inflammatory carcinogens and toxins before they can cause damage and maintain cellular homeostasis [36]. Under stress, Nrf2 may dissociate from its inhibitor Keap-1 and translocate into the cellular nucleus, thereby starting the transcriptional activation pathways of cell defense genes [37]. Several protein kinases, including PKC, ERK, JNK, and p38, modify Nrf2 and activate its release from Keap1/Nrf2 [38]. The upregulation of Nrf2/Keap1 and suppression of NF-κB/MAPK attribute antioxidative, anti-inflammatory, and antiapoptotic effects [39]. Nrf2 interacts with c-Jun and regulates ARE antioxidative gene expressions. Nrf2 also induces NAD (P) H quinone oxidoreductase 1 (NQO1) and catalase (CAT) expression [40]. Nrf2 was correlated with the induction cytoprotective proteins of HO-1, GPx, SOD, and CAT, permitting free radical scavenging in cells caused by oxidative damage [41,42].

In LPS-stimulated RAW 264.7 cells, it was observed that EK100 increased Nrf2 protein expression in the cellular nucleus (Figure 5a). Moreover, EK100 increased HO-1, SOD1, SOD2, and CAT (Figure 5b). The IF staining assay showed that EK100 stimulated the antioxidative transcript factor Nrf2 in the cellular nucleus (Figure 5c)and then promoted the antioxidative proteins of HO-1 expression in the cytoplasm (Figure 5d). Moreover, EK100 also activated the Nrf2/HO-1 signaling pathway in human leukemia monocytic THP-1 cells (Figure 5e) and murine microglial BV2 cells (Figure 5f,g).

Nrf2 siRNA significantly knocked down Nrf2 mRNA and protein levels and elevated intracellular levels of reactive oxygen species (ROS) [43]. Nrf2 siRNA was usually used to knock down the function of Nrf2, HO-1, and relative proteins in RAW 264.7 cells [44]. Simulation results indicate Nrf2 siRNA reversed EK100 promoted the protein overexpression of Nrf2 (Figure 6a), HO-1, SOD1, SOD2, and CAT, respectively (Figure 6b). However, Nrf2 siRNA significantly reversed EK100-induced proteins expressions of the Nrf2/HO-1 signaling pathway (Figure 6c,d). The results in Figure 5 indicated that EK100 induced the transcription factor Nrf2 translocated to the cellular nucleus. Then Nrf2 activated the antioxidant protein expressions of HO-1, SOD1, SOD2, and CAT in the cellular cytoplasm in cells. In brief, EK100 activated the antioxidative Nrf2/HO-1 signaling pathway in LPS-stimulated macrophage-like cells *in vitro*.

## 5. Conclusions

**Figure 7:**
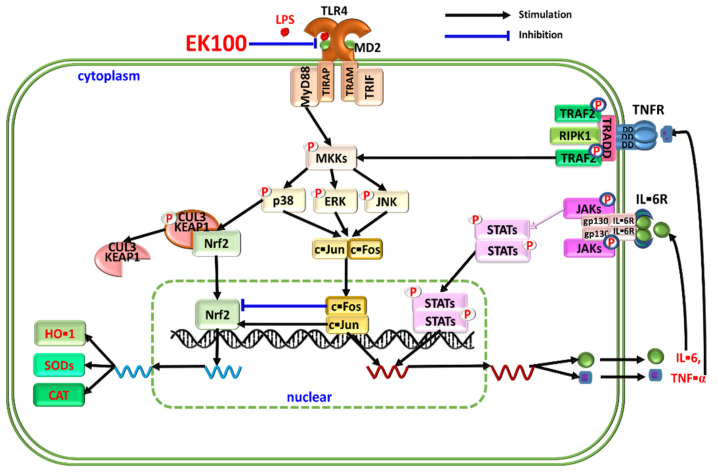
The suggested mechanism relating to the effect of EK100 attenuates inflammatory responses by inhibiting LPS/TLR4 signaling relative MAPK/AP-1 signaling-induced IL-6/JAK/STAT signaling pathway and activating the Nrf2/HO-1 antioxidative signaling pathway in murine macrophage-like RAW 264.7 cells *in vitro*.

This study reveals that EK100 anti-inflammatory effects interfere with the LPS/TLR4 related MAPK/AP-1-induced IL-6/JAKs/STATs inflammatory pathway and activate the Nrf2/HO-1 antioxidative signaling LPS-stimulated macrophage-like cells (Figure 7). The results may lead to approval for EK100 to act as a novel dual strategy through interferences with the inflammatory transcription factor signaling pathway and activate the antioxidative transcription factor signaling pathway to treat inflammatory diseases in the future.

## Data Availability

Data is contained within the article.

## Abbreviations

AP-1	Activator protein-1
AFM	Atomic Force Microscopy
ARE	Antioxidant responsive element
Akt	Protein kinase B
BSA	Bovine serum albumin
CAT	Catalase
CLI-095	Resatorvid; TAK-242
CM	*Cordyceps militaris*
COX-2	Cyclooxygenase-2
Dexa	Dexamethasone
DAPI	4′,6-diamidino-2-phenylindole
DMSO	Dimethyl sulfoxide
DMEM	Dulbecco’s Modified Eagle Medium
ECL	Enhanced chemiluminescence
ELISA	Enzyme-linked immunoassay
ERK	Extracellular-signal-regulated kinase
EK100	Ergosta-7,9(11),22-trien-3β-ol
FBS	Fetal bovine serum
GPx	Glutathione peroxidase
HO-1	Heme oxygenase-1
IF	Immunofluorescence
IL	Interleukin
iNOS	Inducible nitric oxide synthase
IκB	Inhibitor kappa B
IKK	IκB kinase
JAK	Janus kinase
JNK	c-Jun N-terminal kinase
Keap-1	Kelch-like ECH-associated protein
LPS	Lipopolysaccharide
MAPK	Mitogen-activated protein kinase
MD-2	Myeloid differentiation-2
MMP	Matrix metalloproteinase
MTT	3-(4,5-Dimethylthiazol-2-yl)-2,5-diphenyltetrazolium bromide
NF-κB	Nuclear factor-κB
NO	Nitric oxide
NQO1	NAD (P) H quinone oxidoreductase 1
Nrf2 siRNA	Nrf2 Small interfering RNA
Nrf2	Nuclear transcription factor erythroid 2-related factor 2
p38	p38 MAPK
p50	NF-κB p50
p65	NF-κB p65
PI3K	Phosphatidylinositol-3-kinase
PMA	Phorbol 12-myristate 13-acetate
PVDF	Polyvinylidene fluoride
Q-PCR	Quantitative real-time polymerase chain reaction
SDS-PAGE	Sodium dodecyl sulfate-polyacrylamide gel
SOD	Super oxidative dismutases
STAT	Signal transducer and activator of transcription
TLR4	Toll-like receptor 4
TNF-α	Tumor necrosis factor-α
